# Research on Natural Fiber Microstructure Detection Method Based on CA-DeepLabv3+

**DOI:** 10.3390/ma17235942

**Published:** 2024-12-04

**Authors:** Shuaishuai Lv, Xiaoyuan Li, Hitoshi Takagi, Zhengjie Hou, Yifei Zhai, Linfei Chen, Hongjun Ni

**Affiliations:** 1 School of Mechanical Engineering, Nantong University, Nantong 226019, China; lvshuaishuai@ntu.edu.cn (S.L.); 2210310046@stmail.ntu.edu.cn (X.L.); hangtang19870816@126.com (Z.H.); ayfajr@163.com (Y.Z.); 2 Graduate School of Technology, Industrial and Social Sciences, Tokushima University, Tokushima 770-8506, Japan; takagi@tokushima-u.ac.jp; 3 School of Intelligent Manufacturing, Nantong Institute of Technology, Nantong 226019, China

**Keywords:** natural fibers, fiber/matrix bonding, deep learning, DeepLabv3+, EMA mechanism

## Abstract

Natural fibers exhibit noticeable variations in their cross-sections, and measurements assuming a circular cross-section can lead to errors in the values of their properties. Providing more accurate geometric information of fiber cross-sections is a key challenge. Based on microscopic images of natural fiber structures, this paper proposes a natural fiber microstructure detection method based on the CA-DeepLabv3+ network model. The study investigates a natural fiber microstructure image segmentation algorithm that uses MobileNetV2 as the feature extraction backbone network, optimizes the Atrous Spatial Pyramid Pooling (ASPP) module through cascading, and embeds an Efficient Multi-scale Attention (EMA) mechanism. The results show that the algorithm proposed in this paper can accurately segment the microstructures of multiple types of natural fibers, achieving an average pixel accuracy (mPA) of 95.2% and a mean Intersection over Union (mIoU) of 90.7%.

## 1. Introduction

Synthetic fibers, such as glass fibers, carbon fibers, and aramid fibers, are the reinforcing materials for most polymer composites currently produced [1]. However, environmental concerns associated with synthetic fibers and uncertainties in oil prices have prompted in-depth research into the suitability of natural fibers as reinforcing materials. Natural fibers are of great value in industrial applications. In the textile industry, accurate measurement of fiber cross-sectional characteristics can significantly improve the accuracy of fiber grading and enable real-time monitoring of production line quality. In the construction field, accurate fiber geometric characteristics help predict the mechanical properties of natural fiber-reinforced concrete. In composites manufacturing, the method enables online inspection of fiber distribution and alignment to improve product quality stability. Research results indicate that natural fiber-reinforced composites show great potential in multiple fields, including automotive interior components [2] and construction materials [3]. Panels made from natural fiber-reinforced composites not only possess considerable strength and lightweight characteristics, but the materials are also renewable, meeting the requirements of modern sustainable development [4].

Fiber-reinforced composites are primarily composed of two parts: the fibers and the matrix [5]. The fibers provide strength and stiffness to the material, while the matrix binds the fibers together and protects them. The connecting area between the fiber and the matrix is called the interface, which is crucial to the performance of the composite [6]. The interface acts like a “glue” between the fiber and the matrix, helping to transfer forces and enabling the entire material to withstand external pressures [7]. The load bearing capacity and stiffness of the fibers, along with the shear strength of the interface, play key roles in controlling the mechanical properties of the composite [8]. The tensile strength and stiffness values of the fibers depend on the cross-sectional area of the fiber, while the interfacial shear strength value depends on the interfacial area. The interfacial area is the product of the fiber cross-section perimeter and the fiber embedded length, assuming the fiber has a uniform cross-section along its embedded length [9]. Therefore, accurately measuring the area and perimeter of the fiber cross-section provides necessary theoretical support for using natural fibers as reinforcing materials in composites [10].

However, the cross-sectional shape and dimensions of natural fibers vary greatly, making accurate measurement of their area and perimeter complex. Many scholars adopt simplified methods, assuming fibers are cylindrical for calculations [11]. In recent years, with technological advancements, researchers have begun using more sophisticated methods to precisely measure the cross-sectional characteristics of natural fibers, such as Scanning Electron Microscopy (SEM) [12] and X-ray Micro-Computed Tomography (Micro-CT) [13]. These techniques can provide detailed three-dimensional structural information of fibers at the nanoscale, greatly improving measurement accuracy. However, the equipment is very expensive and requires operation by specialized personnel. Consequently, in many studies on natural fibers, it is still common to use average diameter to characterize fiber dimensions [14]. While this simplification is convenient, it may not fully reflect the true geometric characteristics of natural fibers, especially considering the complex structures revealed by advanced measurement techniques.

This paper investigates a natural fiber microstructure detection method based on deep learning, introducing an attention mechanism into the DeepLabv3+ framework and optimizing the ASPP module through cascading. The main objective is to develop an accurate and automated method for natural fiber microstructure detection and measurement. Current approaches often simplify fiber cross-sections as circular shapes, which can lead to significant measurement errors. Natural fiber microstructure images were obtained using scanning electron microscopy, and the model was trained to segment these microscopic structure images. By comparing segmentation results from different deep learning network models, the study verified its excellent performance in natural fiber microstructure image segmentation. This approach improved the accuracy of natural fiber microstructure image extraction, laying a foundation for enhancing the precision of measuring geometric properties such as cross-sectional area, perimeter, and degree of irregularity (DOI) [15] of natural fibers.

## 2. Natural Fiber Microstructure Segmentation Network Based on CA-DeepLabv3+

### 2.1. Overall Framework of CA-DeepLabv3+ Segmentation Network

**Figure 1:**
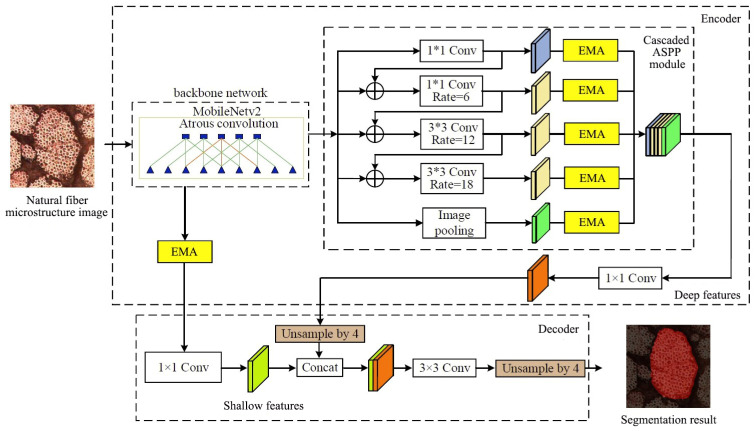
Improved DeepLabv3+ Network Structure.

The Xception backbone network [16] of the standard DeepLabv3+ network improves the accuracy of the network model by expanding network depth and width, but the computational complexity of the Xception backbone network is relatively high, making it difficult to deploy in embedded systems [17]. This paper adopts the MobileNetV2 backbone network [18] to replace the original Xception backbone network, reducing network parameters and improving model running speed. Meanwhile, due to the significant variations in morphology and size of natural fiber microstructures across different images, the original ASPP module in DeepLabv3+ runs branches in parallel, preventing the exchange of microstructure feature information. Therefore, by cascading optimization of the original ASPP module in the DeepLabv3+ network, multi-scale information of the microstructure is extracted and fused progressively, improving the accuracy and robustness of microstructure feature extraction. To address the interference caused by the complex network structure of natural fibers, the interweaving features between fibers, and surface morphology changes during natural fiber microstructure segmentation, an EMA mechanism is embedded in the DeepLabv3+ network model. This fully utilizes multi-scale information, enhances the extraction of deep and shallow features, and improves the ability to extract features from natural fiber microstructure images. The CA-DeepLabv3+ network structure is shown in Figure 1, where ⊕ represents the concatenation operation.

Based on the overall framework description, it is essential to analyze the advantages and disadvantages of different semantic segmentation methods for natural fiber microstructure detection. Table 1 presents a comprehensive comparison of these methods from the perspective of their core principles and applicability to fiber analysis.

According to this comparison, there are several aspects that need to be improved for better natural fiber microstructure detection. The following sections will detail our proposed enhancements to address these limitations.

### 2.2. Selection of Backbone Network

As the original backbone network Xception in DeepLabv3+ has a complex structure and a large number of parameters [19], it may lose fine structures and surface features of fibers when extracting details from complex and variable targets like natural fiber microstructures, leading to inaccurate or discontinuous segmentation results. The microstructure of natural fibers typically presents a complex network morphology, with interweaving and overlapping between fibers, and significant variations in surface morphology [14]. By replacing the original DeepLabv3+ backbone network Xception with the lightweight MobileNetV2, the improved network can extract features from natural fiber microscopic images more quickly. Simultaneously, it enables the model to more easily capture fiber details, interwoven structures, and surface morphologies. This improvement helps to enhance the accuracy and efficiency of natural fiber microstructure segmentation, providing a more reliable foundation for subsequent fiber performance analysis.

**Figure 2:**
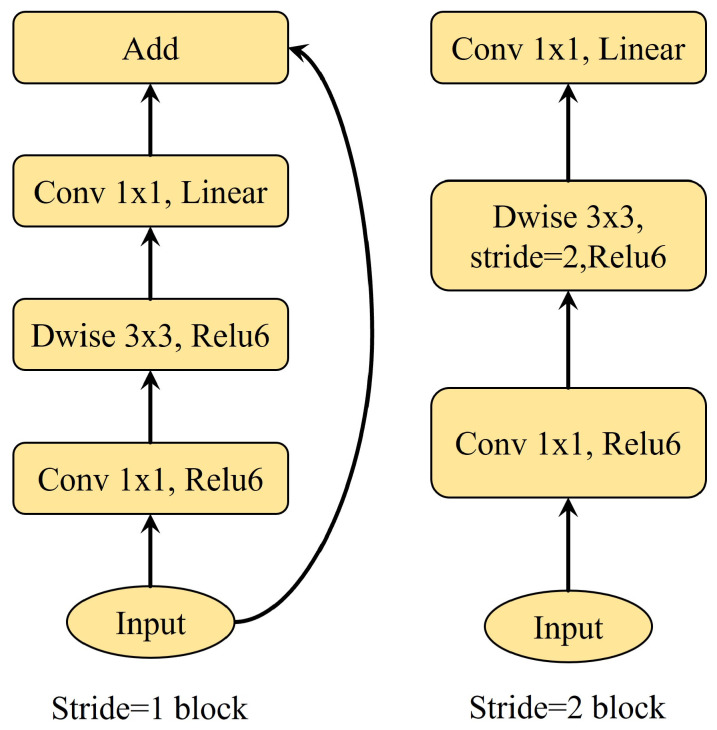
MobileNetV2 Model.

MobileNetV2 is a lightweight convolutional neural network based on the Inverted ResBlock, which reduces model parameters while maintaining model segmentation accuracy [20]. The Inverted ResBlock is divided into two parts. The main part first uses a 1 × 1 convolution operation to increase the dimensionality of features, then uses a 3 × 3 depthwise separable convolution to extract features, and finally uses another 1 × 1 convolution for dimensionality reduction. The residual edge, as the second part, directly connects the input to the output. The MobileNetV2 model is shown in Figure 2.

### 2.3. Cascading Optimization of ASPP Module

In the original ASPP module of DeepLabv3+, branches run in parallel, making it difficult to fully capture the multi-scale features and complex spatial relationships of natural fiber microstructures. By fusing feature layers between different branches, each branch can share multi-level information of the fiber structure, effectively expanding the receptive field of the network model. This allows the network to better extract complex structural and morphological information from natural fiber images. The Receptive Field (*RF*) refers to the size of the input image region corresponding to an output unit in a convolutional neural network, affecting the model’s ability to capture multi-scale features [21]. The calculation of the receptive field is shown in Equation (1):

(1) $$RF=\left(E_{r}-1\right)\times \left(k-1\right)+k$$

where *E_r_* represents the dilation rate, and k represents the size of the convolution kernel.

According to Equation (2), the maximum receptive field *RF*_max_ can be obtained as:

(2) $$RF_{\max}=\max(RF_{3}^{6},RF_{3}^{12},RF_{3}^{18})=RF_{3}^{18}$$

where ${RF\;}_{3}^{6}$ represents the receptive field of dilated convolution when the convolution kernel size is 3 × 3 and the dilation rate is 6.

By connecting the feature layers in the ASPP module, the model can obtain a larger receptive field [22], thereby improving the model’s utilization of complex structural information in natural fiber images and ultimately enhancing the overall accuracy of fiber structure recognition. Specifically, for natural fibers such as flax, hemp, or bamboo fibers, their microstructures often exhibit high irregularity and variability [23]. The improved model can better adapt to this complexity, capturing subtle features of the fibers, such as microfibril angles, changes in fiber cross-sectional shapes, and the layered structure of cell walls. These features are crucial for understanding and predicting the mechanical properties and interface characteristics of the fibers.

### 2.4. Efficient Multi-Scale Attention Mechanism

**Figure 3:**
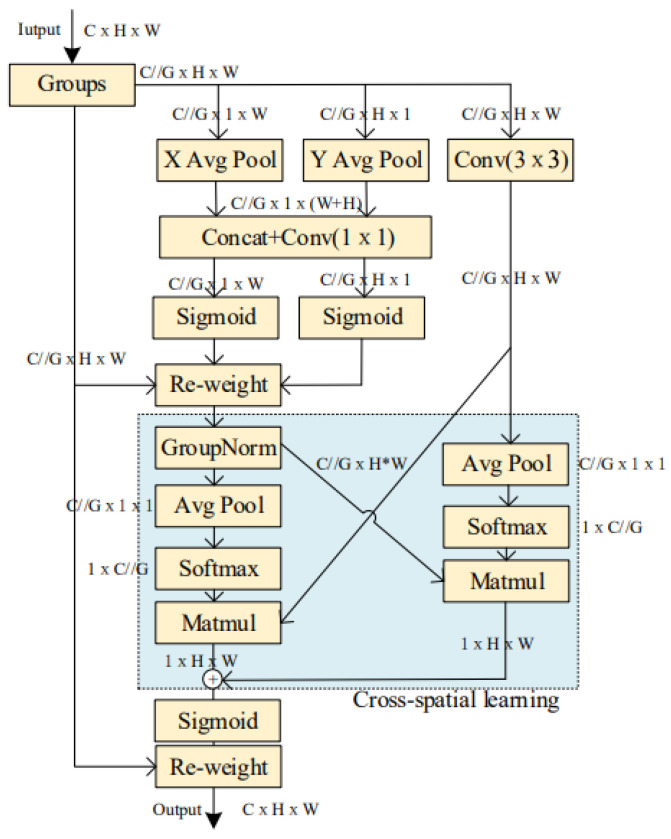
Structure Diagram of EMA Module.

To enhance the feature extraction capability of deep neural networks for natural fiber microstructures and suppress background noise, an Efficient Multi-scale Attention (EMA) mechanism for cross-spatial learning [24] was integrated into the DeepLabv3+ framework. This mechanism significantly improves the model’s feature expression ability and spatial perception accuracy. The EMA module uses parallel sub-structures to reduce network depth, achieving fine pixel-level attention allocation to feature maps without reducing channel dimensions [19]. The structure of the EMA module is shown in Figure 3.

In the EMA module, the input feature map $X\in R^{C\times H\times W}$ is first grouped and then processed through different branches. Two parallel branches perform one-dimensional global average pooling operations on channels along two spatial directions through 1 × 1 convolutions. After processing with the Sigmoid activation function, the two channel attention maps are aggregated together using matrix dot product operations. The third branch uses 3 × 3 convolution to capture multi-scale features. Subsequently, for the two tensors output by the 1 × 1 branch and the 3 × 3 branch, two-dimensional global average pooling is used to encode global spatial information for the output of the 1 × 1 branch. Before the channel feature aggregation activation mechanism, the output of the smallest branch is directly transformed into the corresponding dimensional shape, namely $R_{1}^{1\times C//G}\times R_{3}^{C//G\times HW}$ [25]. Similarly, the dimensional shape of the output from the 3 × 3 branch is $R_{3}^{1\times C//G}\times R_{1}^{C//G\times HW}$.

Finally, the output feature maps within each group are added to generate a set of two spatial attention weights. These are then used with a Sigmoid function and simple multiplication operations to make the final output of EMA the same size as the input feature map [26]. The EMA mechanism enhances the model’s ability to focus on target features, enabling it to obtain more microstructural feature information of natural fibers.

This improvement is particularly effective for analyzing the complex microstructures of natural fibers. Natural fibers, such as flax, hemp, or bamboo fibers, typically exhibit multi-scale structural features. The multi-scale processing capability of the EMA module can simultaneously capture structural information at these different scales, such as the cross-sectional shape of fibers, the layered structure of cell walls, and pore distribution.

## 3. Experimental Analysis

### 3.1. Dataset and Environment Configuration

To train the CA-DeepLabv3+ image segmentation model, it was first necessary to establish an image dataset. The dataset includes microscopic structure images of Bamboo Fiber, Hemp fiber, and Abaca fiber captured by an Olympus BX61 (Shinjuku City, Japan) motorized microscope using the Olympus Stream image analysis software (version 1.9.4). Initially, 500 original images were collected. To expand the dataset to 3500 images, data augmentation operations including flipping, rotation, translation, brightness adjustment, noise addition, mirroring, and stitching were performed using Python’s OpenCV library (version 4.7.0) and imgaug library (version 0.4.0).

**Figure 4:**
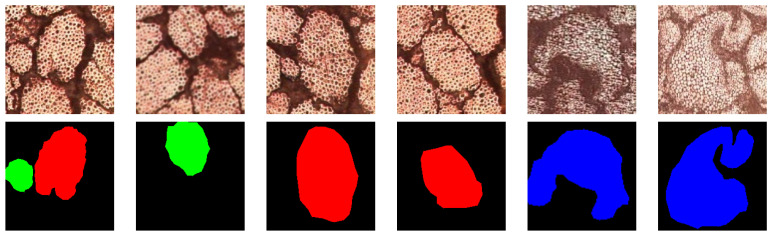
Examples of labeled natural fiber microstructure images and their corresponding mask images.

Subsequently, the Labelme 3.16.7 annotation software was used to label the natural fiber microstructures and generate mask images. Some examples of labeled natural fiber microstructure images and their corresponding mask images are shown in Figure 4. Finally, the augmented natural fiber microstructure images were randomly divided into training and validation sets at a ratio of 9:1.

The experimental environment is as follows: The operating system is Windows 11; GPU is NVIDIA GeForce RTX 3060 Laptop GPU with CUDA parallel computing platform for GPU programming acceleration; CPU is 11th Gen Intel(R) Core (TM) i7-11800H @ 2.30 GHz with 32 GB memory. Python version 3.10 is used for programming; PyCharm Community Edition 2023 is used for network deployment and debugging; the deep learning framework used is Pytorch 2.0.1, with CUDA Version 11.7. The Input_shape is 512 × 512; Batch_size is 8, Num_workers is 4, Epoch is 300, and the initial learning rate is 0.007.

### 3.2. Experimental Comparison

**Figure 5:**
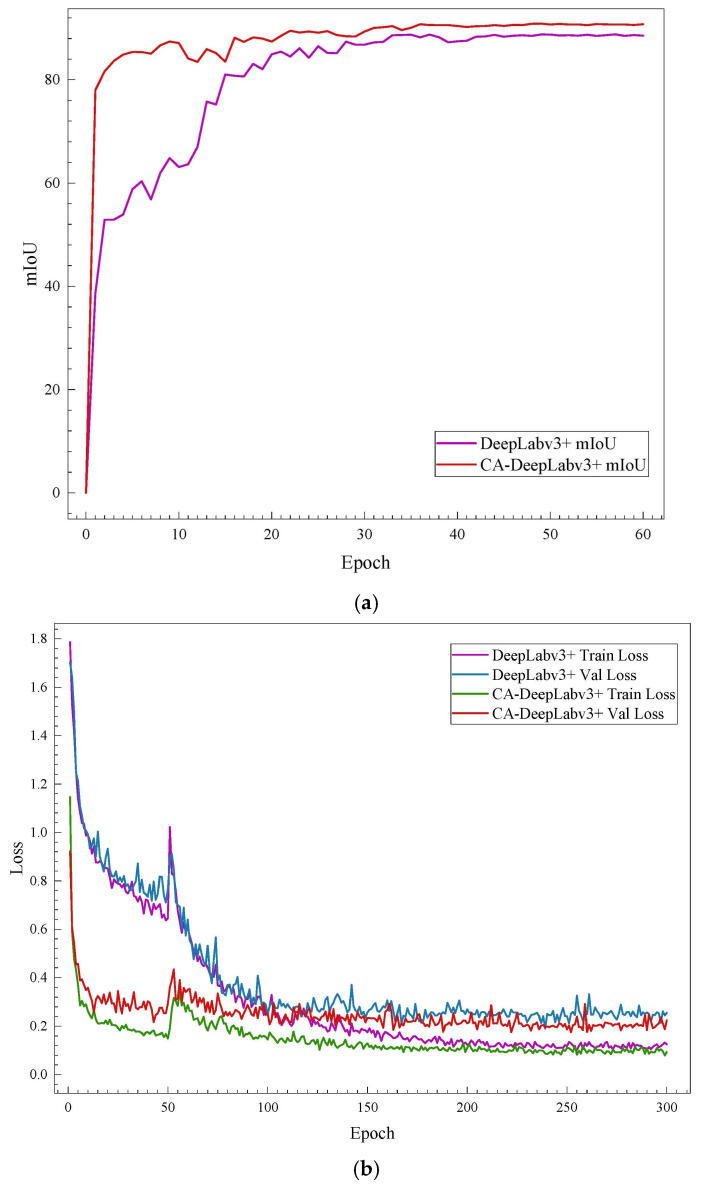
Visualization comparison of performance indicators for different networks. (**a**) Visualization comparison of average Intersection over Union (MIoU) results. (**b**) Visualization comparison of loss function results.

To verify the superiority of the proposed natural fiber microstructure detection method based on improved DeepLabv3+, this paper compares the segmentation performance indicators of the proposed CA-DeepLabv3+ algorithm with current mainstream image segmentation algorithms such as U-Net, PSPNet (Pyramid Scene Parsing Network), and standard DeepLabv3+ network models. Under the same training parameter conditions, the comparison of segmentation performance indicators for microstructures by different methods is shown in Table 2. During the training of different networks, the mIoU is recorded every five epochs, and the visualization comparison of training results for different networks is shown in Figure 5.

The CA-DeepLabv3+ network model proposed in this paper outperforms the comparison algorithms in both segmentation accuracy and detection rate. Specifically, compared to the standard DeepLabv3+, U-Net, and PSPNet network models, the detection precision increased by 1.36, 10.50, and 17.85 percentage points, respectively, while the recall rate improved by 0.93, 17.45, and 21.82 percentage points, respectively. The mPA improved by 0.9%, 17.5%, and 21.7%, respectively, and the mIoU increased by 2.1%, 18.5%, and 23.9%, respectively. Furthermore, while the algorithm proposed in this paper achieves more precise segmentation of natural fiber microstructure images, it also consistently shows higher F1 scores compared to the standard DeepLabv3+, U-Net, and PSPNet network models, and can meet real-time detection requirements.

**Figure 6:**
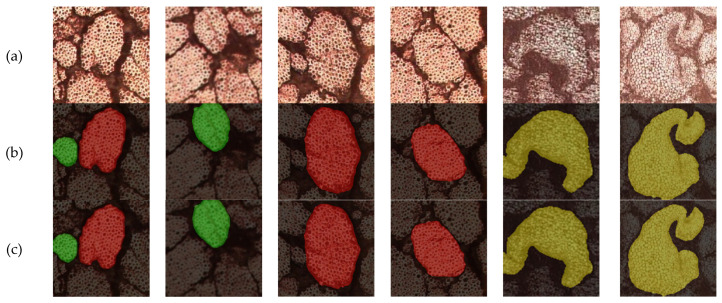
Prediction results of different networks. (**a**) Original microscopic images of natural fibers; (**b**) Segmentation results using standard DeepLabv3+; (**c**) Results from improved CA-DeepLabv3+.

To verify the reliability and detection accuracy of the proposed method, a comparison experiment on detection accuracy was conducted. After training different networks, predictions were made on the test set. Using the six natural fiber microstructure images in Figure 6a as part of the test set, predictions were made using both the DeepLabv3+ network model and the CE-DeepLabv3+ network model proposed in this paper. The visualization results of the microstructure predictions from different networks are shown in Figure 6b,c, respectively.

As shown in Figure 6, the CA-DeepLabv3+ algorithm achieves superior performance in natural fiber segmentation. The original images (a) display three different types of natural fibers with varying morphologies and structural arrangements. Compared to standard DeepLabv3+ results (b), which show jagged edges and unclear boundaries in overlapping regions, our improved CA-DeepLabv3+ (c) demonstrates smoother edge definition, clearer boundary delineation, and better preservation of morphological details. The algorithm maintains robust segmentation performance even with complex fiber structures and backgrounds.

## 4. Conclusions

This study proposes a natural fiber microstructure detection method based on CA-DeepLabv3+. To address the complex microstructural features of natural fibers, the following improvements were made to the original DeepLabv3+ network:

(1) MobileNetV2 was adopted as the feature extraction backbone network, reducing computational complexity and improving model running speed;
(2) The Atrous Spatial Pyramid Pooling (ASPP) module was optimized through cascading, enhancing the model’s ability to extract multi-scale features of natural fibers;
(3) An Efficient Multi-scale Attention (EMA) mechanism was embedded, improving the model’s perception of microstructural details in natural fibers;
(4) Compared to mainstream image segmentation algorithms such as standard DeepLabv3+, U-Net, and PSPNet, the CA-DeepLabv3+ algorithm shows significant advantages in natural fiber microstructure detection: detection precision improved by 1.36–17.85%, recall improved by 0.93–21.82%, mean pixel accuracy (mPA) improved by 0.9–21.7%, mean Intersection over Union (mIoU) improved by 2.1–23.9%, and F1 scored consistently higher than comparison algorithms.

The CA-DeepLabv3+ algorithm not only improves the segmentation accuracy of natural fiber microstructures but also better handles fiber edges in complex backgrounds while maintaining high detection efficiency. This method provides a reliable foundation for accurately measuring geometric properties such as cross-sectional area, perimeter, and DOI of natural fibers. It is of significant importance for in-depth understanding of the microstructural characteristics of natural fibers and their relationship with macroscopic properties.

## Figures and Tables

**Table 1:** Comparison of different methods for natural fiber microstructure detection.

Method	Advantages	Disadvantages
CA-DeepLabv3+	Enhanced multi-scale feature extraction through cascaded ASPP module Improved fiber detail and morphological feature perception via EMA mechanism Reduced computational complexity with lightweight MobileNetV2 Excellent handling of complex fiber interweaving and overlapping scenarios	Relatively complex multi-module structure after improvements Requires substantial annotated data for network training Relatively longer model training time
Standard DeepLabv3+	Multi-scale information capture through ASPP module Expanded receptive field via atrous convolution	High computational cost and complexity Limited feature information interaction Insufficient fiber detail extraction
U-Net	Simple encoder–decoder architecture Spatial information preservation through skip connections	Lack of dedicated multi-scale extraction mechanism Poor complex fiber structure processing Limited fiber detail perception
PSPNet	Global context capture through pyramid pooling Straightforward architecture implementation	Insufficient attention to local fiber details Basic feature fusion approach Poor fine fiber structure segmentation

**Table 2:** Comparison of segmentation performance for different methods.

Model	Detection Results				Training Time	F1/%
	mIoU	mPA	Precision	Recall		
CA-DeepLabv3+	0.907	0.952	0.9488	0.9520	44h	95.1
DeepLabv3+	0.886	0.943	0.9352	0.9427	30h	93.9
UNet	0.722	0.777	0.8430	0.7775	26h	80.9
PSPNet	0.668	0.735	0.7703	0.7338	15h	71.5

## Data Availability

The original contributions presented in the study are included in the article, further inquiries can be directed to the corresponding authors.
